# Mechanism of Action of Magnesium Lithospermate B against Aging and Obesity-Induced ER Stress, Insulin Resistance, and Inflammsome Formation in the Liver

**DOI:** 10.3390/molecules23092098

**Published:** 2018-08-21

**Authors:** Ji won Jeong, Bonggi Lee, Dae Hyun Kim, Hyoung Oh Jeong, Kyoung Mi Moon, Min Jo Kim, Takako Yokozawa, Hae Young Chung

**Affiliations:** 1 Molecular Inflammation Research Center for Aging Intervention (MRCA), College of Pharmacy, Pusan National University, Busan 609-735, Korea; na881103@naver.com (J.w.J.); bong3257@gmail.com (B.L.); bioimmune@hanmail.net (D.H.K.); gagagod84@gmail.com (H.O.J.); omkksm@nate.com (K.M.M.); kiki10304@gmail.com (M.J.K.); 2 Coreana Cosmetics Co., Ltd., Cheonan-si, Chungcheongnam-do 330-833, Korea; 3 Korean Medicine (KM)-Application Center, Korea Institute of Oriental Medicine (KIOM), 70 Cheomdan-ro, Dong-gu, Daegu 41062, Korea; 4 Graduate School of Science and Engineering for Research, University of Toyama, Toyama 930-8555, Japan; yokozawa@inm.u-toyama.ac.jp

**Keywords:** magnesium lithospermate B, PPARβ/δ, ER stress, insulin resistance, inflammasome, aging

## Abstract

Magnesium lithospermate B (MLB) is the biologically active compound of the water-soluble fraction of *Salvia miltiorrhiza*. Magnesium lithospermate B exhibits various biological functions, including antidiabetic, neuroprotective, and antioxidant effects. However, its beneficial effects on insulin sensitivity and related signaling pathways in the liver need to be elucidated. Our previous study reported that MLB is a PPARβ/δ agonist in fibroblasts. Because insulin-sensitizing and anti-inflammatory effects of PPARβ/δ has been reported in the liver, we investigated whether MLB has a beneficial effect on insulin-, ER stress- and inflammasome-related signaling in the livers of aging and obese animal models. Western blotting and protein-ligand docking simulation showed that MLB activated PPARβ/δ and improved glucose tolerance in the livers of aging and obese animal models. MLB supplementation ameliorated aging or obesity-induced disruption of insulin signaling in the liver. Consistently, aging and obesity-induced increase in the protein levels of a gluconeogenic phosphoenolpyruvate carboxykinase was decreased by MLB. When molecular signaling pathways related to insulin signaling were examined in the liver, MLB supplementation suppressed ER stress- and inflammasome-related signaling molecules induced by aging and obesity. These results suggest that MLB may improve insulin resistance in the liver at least partially by suppressing ER stress and inflammasome formation in aging and obese animal models.

## 1. Introduction

Aging represents the accumulation of changes in a human being over time, encompassing physical, psychological, and social changes. Age-related diseases include diabetes, cancer, arthritis, dementia, vascular diseases, obesity, and metabolic syndrome [1]. Today, the aging population has an increased incidence of diabetes and obesity, which are closely associated with insulin resistance [2].

Obesity is a medical condition with excessive body fat accumulation caused by increased food intake and/or lack of energy expenditure. According to the World Health Organization, at least 1 in 10 adults worldwide are obese. Obesity is closely associated with metabolic syndrome, leading to reduced life span by increasing a risk of various diseases including stroke, diabetes, cardiovascular diseases, and cancers [3]. It is one of the most serious public health problems of the 21st century associated with aging [4]. Therefore, managing obesity-related metabolic syndrome including insulin resistance, dyslipidemia, and fatty liver may be an important strategy to ameliorate aging-related disorders.

Insulin resistance is a pathological condition in which cells fail to respond to the normal signal of insulin to store glucose in the tissues. As a result, hyperglycemia and hyperinsulinemia occur because of less glucose uptake from metabolic tissues in response to insulin and the triggering of pancreatic beta cells to secrete more insulin to control glucose homeostasis. Increased endoplasmic reticulum (ER) stress and inflammasomes during the aging process also play an important role in insulin resistance [5,6,7,8,9].

The ER is an organelle that regulates cellular calcium storage, protein synthesis, and protein folding. An accumulation of unfolded or misfolded proteins in the lumen of the ER induces an ER stress response or unfolded protein response (UPR) that has been related to diverse metabolic or aging-related diseases [10,11]. The main UPR signaling cascades are initiated by three ER-localized protein sensors: inositol-requiring enzyme 1 (IRE1), protein kinase RNA-like endoplasmic reticulum kinase (PERK), and activating transcription factor 6α (ATF6α). IRE1 activated by ER stress stimulates c-Jun N-terminal kinase (JNK) activation. Subsequently, JNK phosphorylates serine residues of IRS-1, thereby inhibiting insulin receptor signaling [12].

A recent study showed the relationship between inflammasomes and ER stress. ER stress inducers activate NACHT, LRR and PYD domains-containing protein 3 (NLRP3) inflammasomes in human and murine macrophages [13]. The inflammasome is a multiprotein oligomer consisting of NLRP, caspase-1, and apoptosis-associated speck-like protein containing a CARD (ASC). It triggers the activation of inflammatory caspase-1 and the production of interleukin 1β (IL-1β) and interleukin 18 (IL-18). The inflammasome, which is activated by ER stress, alters insulin sensitivity and is activated during inflammation, causing insulin resistance [14,15,16].

The peroxisome proliferator-activated receptors (PPARs) belong to the nuclear hormone receptor superfamily, which is ligand-modulated transcription factors. PPARs heterodimerize with retinoid X receptors (RXRs) and bind to PPAR response elements (PPRE), in the promoter region of their target genes, regulating the transcription of target genes. Currently, three different subtypes, PPARα (NR1C1), PPARβ/δ (NR1C2), and PPARγ (NR1C3) have been identified. Among them, PPARβ/δ is expressed in organs/cells in relatively high levels in metabolically active tissues [17,18,19,20,21,22,23,24,25,26].

*Salvia miltiorrhiza*, belonging to the family Labiatae, is a well-known traditional Chinese herb that has been used to treat various pathologic conditions because of its excellent medicinal properties. Among its diverse functional components, magnesium lithospermate B (MLB) is a major active component from *Salvia miltiorrhiza* with free radical-scavenging, hypotensive, renal function-improving, and anti-skin aging properties [27,28,29,30]. Although MLB has exhibited multiple effects including antioxidant and anti-skin aging functions, the antidiabetic effect of MLB needs to be elucidated. The liver plays a distinct role in the control of insulin sensitivity and glucose homeostasis by regulating blood glucose. Although various signaling pathways are related to hepatic insulin sensitivity, it has been reported that ER stress-induced inflammasome activation contributes to hepatic inflammation and steatosis [31], which are major contributors to hepatic insulin resistance. In addition, studies indicated that hepatic ER stress, inflammation, and steatosis are closely associated with whole-body glucose homeostasis in obesity [31,32,33,34]. Because MLB has been shown to plays a protective role in liver injury and inflammation [35], we investigated the effect of MLB on molecular factors associated with hepatic insulin resistance including ER stress and inflammasomes. For this study, we used obese and aging animal models because aging and obesity are serious problems over the world and both conditions are closely associated with insulin resistance as mentioned earlier.

## 2. Results and Discussion

### 2.1. Activation of PPARβ/δ by MLB in Glucose Metabolism

PPARβ/δ has been shown to decrease ER stress, insulin resistance, and inflammasome formation [36,37,38,39], which are upregulated during aging and obesity. Our previous study reported that MLB is a potential PPARβ/δ agonist in fibroblasts [40]. However, it is unclear whether MLB also stimulates PPARβ/δ activation in the liver in aging and/or obese conditions.

**Figure 1:**
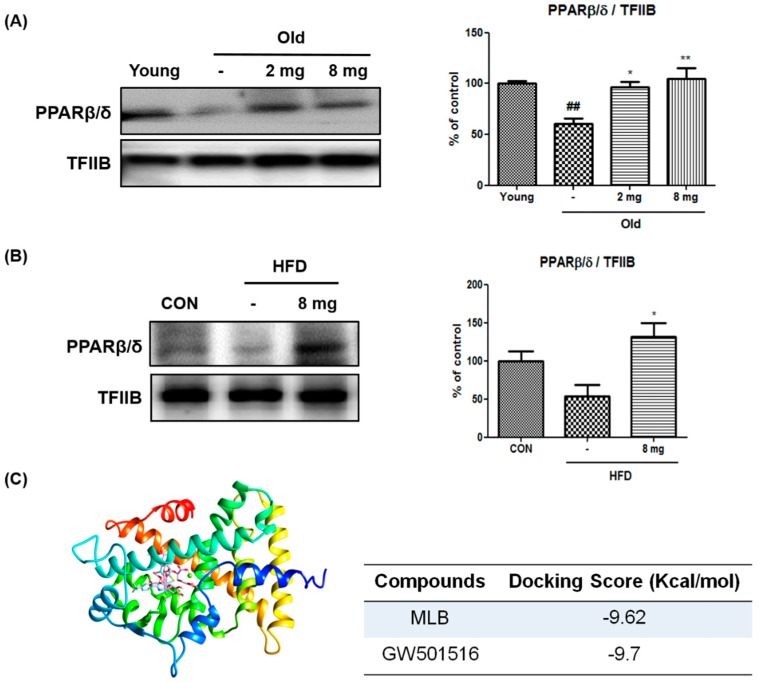
Effect of MLB on PPARβ/δ activation. (**A**,**B**) The level of PPARβ/δ was analyzed by western blotting (*n* = 4 per group). The nucleus protein level of PPARβ/δ was normalized by transcription factor II B (TFIIB). Data represents the mean ± S.E.M. Statistical results of one-way analysis of variance (ANOVA): ^##^ *p* < 0.01 versus the young rats, * *p* < 0.05, ** *p* < 0.01 versus the MLB-untreated aged rats and HFD-fed mice. 2 mg, MLB 2 mg/kg/day; 8 mg, MLB 8 mg/kg/day; HFD, high-fat diet-fed mice. (**C**) Docking simulation was performed to identify the interaction between MLB and the ligand binding domain of human PPARβ/δ. Compound MLB has similar binding sites compared with the known PPARβ/δ agonists GW501516.

To examine this, western blotting was performed on the liver homogenates of aged rats and high-fat diet (HFD)-fed mice that showed increased body weight (Supplementary Figure S1). Because our preliminary studies exhibited that MLB administration for 20–24 days in aging rats or HFD-induced obese mice did not affect body weight (unpublished data), no further analysis related to body weight was performed. Western blotting showed that the protein levels of PPARβ/δ in the nucleus decreased in the livers of aged rats and HFD-fed mice compared to them in young rats and chow diet-fed mice. However, MLB treatment notably increased PPARβ/δ protein levels in the liver nuclei of aged rats and HFD-induced obese mice (Figure 1A,B).

To further test the effect of MLB on PPARβ/δ, the protein-ligand docking simulation was conducted using the Autodock 4.2 program, where MLB linked with 2-bromophenol to provide numerous hydrophobic interactions in the binding pocket. MLB had a binding affinity to the same binding pocket as a known PPARβ/δ ligands, GW501516. In addition, the predicted binding energy of MLB (−9.62 kcal/mol) was similar to that of GW501516 (−9.70 kcal/mol) (Figure 1C). These results suggest that MLB may bind to and activate PPARβ/δ.

**Figure 2:**
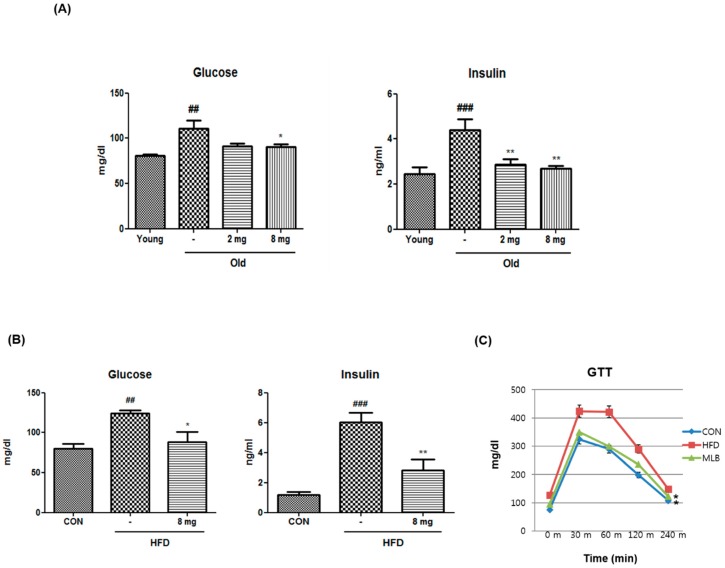
Effect of MLB on metabolic parameters. (**A**) Aged rats were treated for 20 days with MLB (2 or 8 mg/kg/day) and compared with MLB-untreated aged rats. (**B**) HFD-fed mice were treated for 24 days with MLB (8 mg/kg/day). (**C**) Glucose tolerance test following fasting for 18 h in C57BL/6J mice fed a standard chow or high-fat diet. The area under the curve was calculated for the statistics. Data represents the mean ± S.E.M. One-way analysis of variance (ANOVA) was used to determine the statistical result: ^##^ *p* < 0.01, and ^###^ *p* < 0.001 versus the young rats or the HFD-fed mice, * *p* < 0.05, ** *p* < 0.01 versus the MLB-untreated aged rats or HFD-fed mice. 2 mg, MLB 2 mg/kg/day; 8 mg, MLB 8 mg/kg/day; HFD, high-fat diet; GTT, glucose tolerance test.

To investigate the effects of MLB on glucose metabolism, serum glucose and insulin levels were measured in aged rats and HFD-fed mice after overnight fasting. Fasting glucose and insulin levels were markedly increased in aged rats and HFD-fed mice compared to the control groups, and MLB treatment reversed them (Figure 2A,B). Furthermore, a glucose tolerance test showed that HFD impaired glucose tolerance, but this effect was fully reversed by MLB treatment (Figure 2C). Together, the data suggest that MLB ameliorates aging and obesity-induced glucose tolerance.

**Figure 3:**
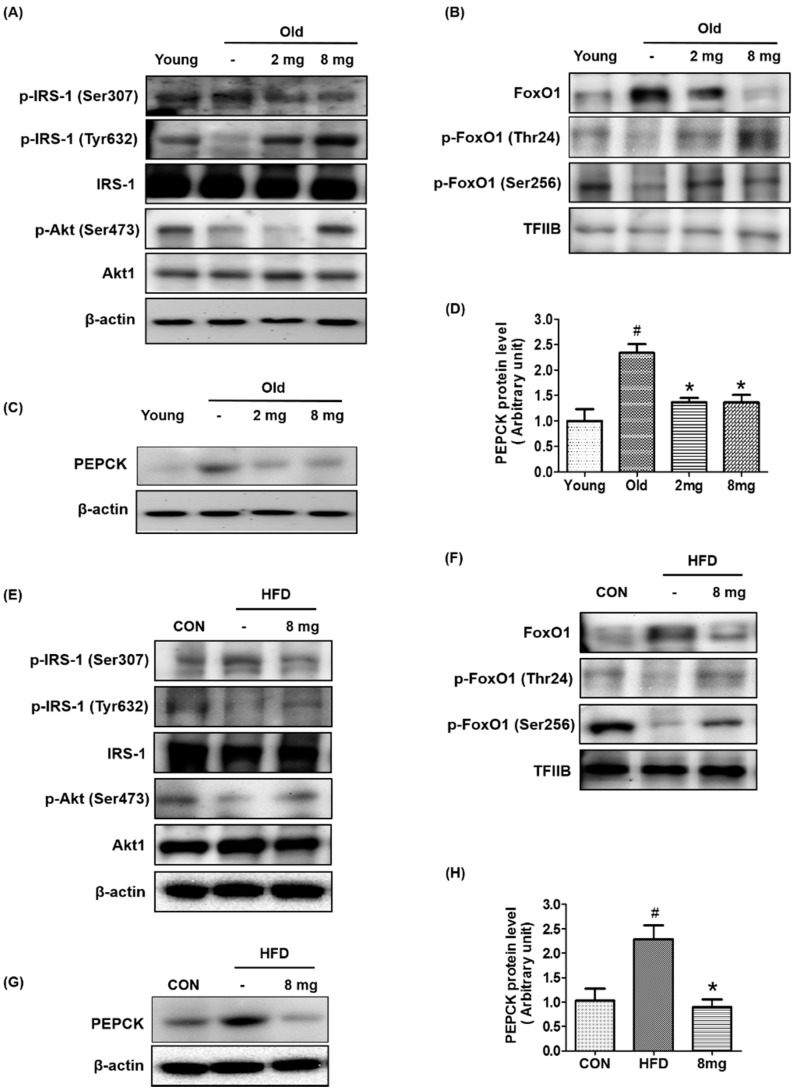
Effect of MLB on the insulin signaling pathway. The protein levels related to insulin signaling were analyzed by western blotting in the liver of (**A**–**C**) aged rat (*n* = 4–5 per group) and (**E**–**G**) HFD-fed mice (*n* = 4–5 per group). (**D**,**H**) The protein level of PEPCK was semi-quantified by the image J software. 2 mg, MLB 2 mg/kg/day; 8 mg, MLB 8 mg/kg/day; Akt, Protein kinase B; FoxO1, Forkhead box protein O1; HFD, high-fat diet; IRS, Insulin receptor substrate; PEPCK, Phosphoenolpyruvate carboxykinase. ^#^ *p* < 0.05 compared to the young or control group and * *p* < 0.05 compared to the old or HFD group.

Because fasting glucose levels are primarily regulated by insulin signaling in the liver, we examined whether MLB regulates insulin signaling in the livers of aging and obese animal models. As shown in Figure 3A,D, p-IRS-1 (Ser307), a marker of insulin resistance, increased in aged rats and HFD-fed mice, whereas MLB treatment reduced it to a level comparable to that of the control group. In parallel with this, aging or HFD-induced decreases in p-IRS-1 (Tyr632) and p-Akt (Ser473) were ameliorated by MLB treatment.

Forkhead box protein O1 (FoxO1) is a transcription factor that is mainly regulated by Akt under insulin signaling. It has three phosphorylation sites controlled by Akt at Thr24, Ser256, and Ser319. Dephosphorylation of FoxO1 enhances FoxO1 stability and transcriptional activity, thereby stimulating gluconeogenesis and hyperglycemia. Phosphorylation of FoxO1 by Akt promotes FoxO1 translocation from the nucleus to the cytoplasm, thereby inhibiting the transcriptional activity of FoxO1 [41]. As shown in Figure 3B,F, the protein level of FoxO1 increased in the livers of aged rats and HFD-fed mice. However, it decreased in the MLB-treated groups (Figure 3B,F). In contrast, the aging and obesity-induced decrease in inactive p-FoxO1 was recovered in the MLB-treated groups (Figure 3B,F). As a downstream gene of FoxO1, we investigated protein expression levels of PEPCK, a key protein for gluconeogenesis in the livers of aging and obese animal models. Western blotting showed that aging or obesity-induced increases in the PEPCK protein level were notably reduced by MLB (Figure 3C,D,G,H). The data indicate that MLB may reduce gluconeogenesis at least partially by modulating Akt/FoxO1 signaling.

### 2.2. Effect of MLB on ER Stress and Inflammasome

**Figure 4:**
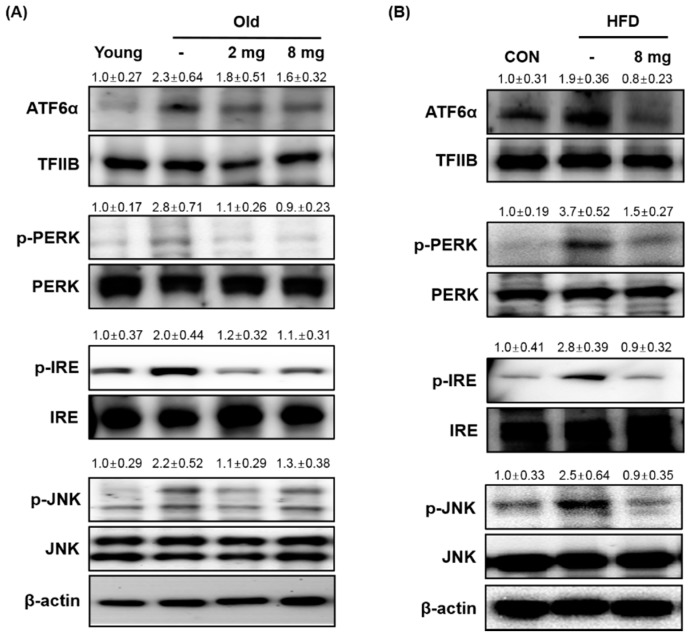
MLB attenuated ER stress. (**A**,**B**) Western blotting was performed to detect the protein level of ER stress markers (*n* = 4–5 per group). Protein levels of ER stress markers decreased in the MLB-treated groups. 2 mg, MLB 2 mg/kg/day; 8 mg, MLB 8 mg/kg/day; ATF6α, activating transcription factor 6α; ER, endoplasmic reticulum; HFD, high-fat diet; IRE, inositol-requiring enzyme 1; JNK, c-Jun N- terminal kinase; PERK, pancreatic ER kinase.

ER stress significantly contributes to the development of insulin resistance by impairing insulin signaling through hyperactivation of JNK followed by Ser307 phosphorylation of IRS-1 [32]. Because Ser307 phosphorylation of IRS-1 was significantly altered by MLB treatment, we investigated whether MLB regulates ER stress in aging and obese conditions by western blotting. The protein levels of ER stress markers including ATF6α, p-PERK, p-IRE, and p-JNK were increased in the aging or obese animal model, but MLB treatment reduced them (Figure 4A,B), suggesting that MLB inhibits aging or obesity-induced ER stress. The ER is particularly susceptible to protein misfolding. Proteins that are unable to fold correctly because of alterations in the physiological and molecular environment cause ER stress and activate the UPR [42], which are closely related to metabolic syndrome in aging and obesity [43], partially due to the disruption of insulin signaling [44,45]. Therefore, chemical or natural compounds that alleviate ER stress may act as potential insulin-sensitizing agents against aging or obesity-induced metabolic syndrome. 

**Figure 5:**
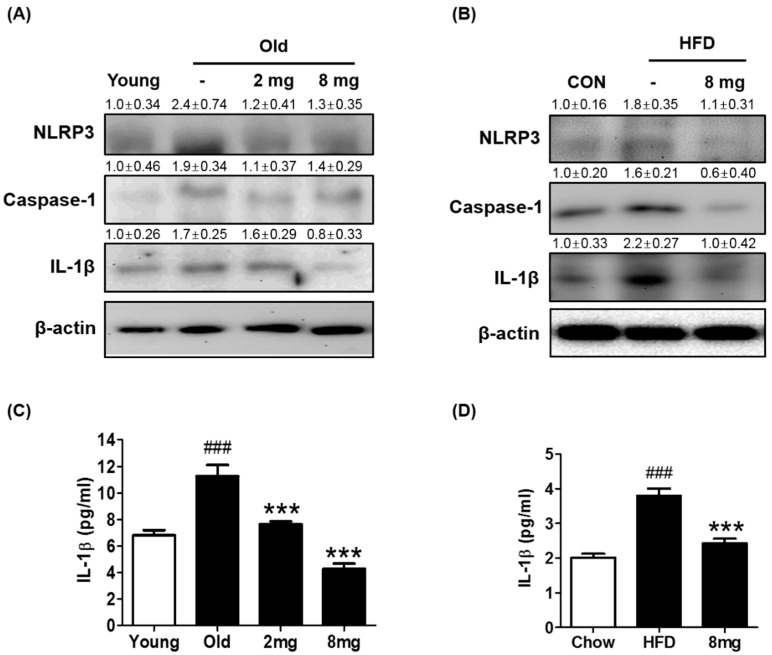
Inhibition of inflammasome formation by MLB. Western blotting was performed to assess inflammasome levels in the aged rat (**A**) and HFD-fed mouse (**B**) livers. The images of western blotting were semi-quantified by the image J software. Serum IL-1β was measured in (**C**) aged rat (*n* = 5 per group) and (**D**) HFD-fed mice (*n* = 7 per group) using the Luminex multiplex analysis system. 2 mg, MLB 2 mg/kg/day; 8 mg, MLB 8 mg/kg/day; HFD, high-fat diet; IL-1β, Interleukin-1β; NLRP3, Pyrin domain containing 3. ^###^ *p* < 0.001 compared to the young or chow group and *** *p* < 0.001 compared to the old or HFD group

Inflammasomes are caspase-1-activating multi-protein complexes, composed of NLRP, caspase-1, and ASC. NLRP recruits the adapter ASC, which in turn recruits procaspase-1. Procaspase-1 autocatalyzes its cleavage and activation, resulting in the maturation of the precursor forms of IL-1β and IL-18 into active proinflammatory cytokines [46]. Because ER stress has been shown to induce inflammasome formation and our data showed that MLB ameliorates ER stress, we examined whether MLB alters inflammasome formation in aging and obesity. Western blotting data showed that protein levels of inflammasome markers including NLRP3, caspase-1, and IL-1β were elevated, but MLB significantly attenuated them (Figure 5A,B). Furthermore, MLB reversed aging- and obesity-induced increase in IL-1β in the blood (Figure 5C,D) indicating that MLB prevents aging or obesity-induced systemic inflammation. The inflammasome is another component that has been shown to inhibit insulin signaling in metabolic disease conditions presumably by inducing inflammation [46]. In addition, various in vitro and in vivo studies showed that ER stress is associated with inflammasome formation. Our study also showed elevated protein levels of inflammasome markers including NLRP3, caspase-1, and IL-1β in aging and obese conditions. However, MLB attenuated them to a level comparable to that of the control group, indicating that MLB-mediated inhibition of inflammasomes is also related to the ameliorated insulin signaling in the liver of the aged or obese animal. Taken together, aging and obesity elevated the pathways of ER stress (ATF6α, p-PERK, p-IRE, and p-JNK) and inflammasome formation (NLRP3, caspase-1, and IL-1β) in the liver (Table 1), which are closely related to the decrease in insulin signaling. MLB administration partially reversed these unfavorable alterations in the liver of aging rats and obese mice (Table 1).

In this study, we orally administered MLB at 2 or 8 mg/kg/day for 20 or 24 days depending on the animal model based on our preliminary experiments and previous publication [47]. This concentration was enough to ameliorate aging-induced renal inflammation and senescence [47]. In the current study, oral administration of MLB at 8 mg/kg/day efficiently ameliorated aging and obesity-induced increase in blood glucose and insulin levels and insulin resistance-related signaling pathways. However, it is unclear whether it is the best concentration to suppress aging or obesity-induced hepatic insulin resistance and related signaling pathways because another concentration range was not tested in this study. 

Although our data showed that MLB ameliorated aging or obesity-induced ER stress and inflammasome formation, contributing to the improvement of insulin signaling in the liver, we did not investigate the lipoprotein levels in the blood. However, it is likely that MLB may affect the lipoprotein secretion from the liver because hepatic insulin resistance is closely associated with dyslipidemia [48]. A study indicates that liver insulin receptor knockout mice, a pure hepatic insulin resistance model, exhibited a proatherogenic lipoprotein profile with decreased HDL and VLDL particles that are notably enriched in cholesterol [48]. Because MLB improved hepatic insulin signaling in aging and obese animal models, it is possible that MLB may have beneficial effects on lipoprotein metabolism. In support of this, it has been shown that MLB supplementation suppressed high-fat diet-induced increase in triglyceride, total cholesterol and elevated HDL levels in the blood of rats [49]. Further studies will be necessary to reveal the roles of MLB in lipoprotein assembly and secretion.

## 3. Materials and Methods

### 3.1. Materials

Magnesium lithospermate B was provided by Professor Takako Yokozawa (Toyama Medical and Pharmaceutical University, Toyama, Japan) and Takashi Tanaka (Nagasaki University, Nagasaki, Japan). The first antibodies used in the western blotting are as follows: PPARβ/δ (Santa Cruz Biotechnology, Santa Cruz, CA, USA/SC-7197), TFIIB (Santa Cruz/SC-271736), p-IRS1(Ser307) (Santa Cruz/SC-33956), p-IRS1(Tyr632) (Santa Cruz/SC-17196), IRS1 (Santa Cruz/SC-559), p-Akt(Ser473) (Santa Cruz/SC-9271), Akt1 (Santa Cruz/SC-1618), β-actin (Santa Cruz/SC-47778), Foxo1 (Santa Cruz/SC-67140), p-Foxo1(Thr24) (Santa Cruz/SC-16561), p-Foxo1(Ser256) (Santa Cruz/SC-10168), PEPCK (Santa Cruz/SC-28477), ATF6α (Santa Cruz/SC-22799), p-PERK(Thr981) (Santa Cruz/SC-32577), PERK (Santa Cruz/SC-13073), p-IRE(Ser274) (Abcam, Cambridge, MA, USA/ab48187), IRE (Santa Cruz/SC-390960), p-JNK (Santa Cruz/SC-6254), JNK (Santa Cruz/SC-7345), NLRP3 (Santa Cruz/SC-34408), Caspase-1 (Santa Cruz/SC-514), and IL-1β (Santa Cruz/SC-1252). The secondary antibodies used are as following; Anti-Rabbit (Genetex, Irvine, CA, USA/GTX213110-01), Anti-Mouse (Genetex/GTX213111-01), and Anti-Goat (Santa Cruz/SC-2768).

### 3.2. Animal Experiments

#### 3.2.1. Experimental Design for Aging Rats

Male Sprague-Dawley (SD) rats (young, aged 5 months and old, aged 20 months) were obtained from Samtako (Osan, South Korea). SD rats groups (*n* = 5/group) were given water and a standard laboratory diet *ad libitum* (AL). MLB was orally administered to the aging rats (the daily dose of MLB was 2 or 8 mg/kg/day for 20 days).

#### 3.2.2. Experimental Design for the Obese Mouse Model

Male C57BL/6J mice (6 weeks of age) were obtained from Samtako. C57BL/6J mice (*n* = 7/group) were fed a chow or a high-fat diet (Research Diet, New Brunswick, NJ, USA, D12492) for 3 months. MLB was orally administered to the obese mice (the daily dose of MLB was 8 mg/kg/day for 24 days). The animal protocol used in this study was reviewed and approved by the Pusan National University-Institutional Animal Care and Use Committee (PNU-IACUC) with respect to the ethical of procedures and scientific care (PNU 2008-0543, PNU-2015-0944).

### 3.3. Tissue Homogenate

All solutions, tubes, and centrifuges were maintained at 0–4 °C. One gram of liver was homogenized with 700 μL of hypotonic lysis buffer [buffer A: 100 mM Tris (pH 7.4), 20 mM β-glycerophosphate, 20 mM NaF, 2 mM sodium orthovanadate, 1 mM EDTA, 0.01 mM DTT, 0.5 mM PMSF, 1 μM pepstatin, 2 μM leupeptin, and 10 mM HEPES (pH 7.8)] using a tissue homogenizer for 30 s. After homogenates were kept on ice for 20 min, 125 μL of 10% Nonidet P-40 (NP-40) solution was added, mixed for 15 s, and then centrifuged at 12,000× *g* at 4 °C for 15 min. The supernatants were used as the cytosol fraction. The pellets were washed with 300 μL of hypotonic buffer A plus 25 μL of 10% NP-40, centrifuged, suspended in 200 μL of buffer C [50 mM KCl, 300 mM NaCl, 1 mM dithiothreitol (DTT), 0.1 mM EDTA, 0.1 mM PMSF, 10% (*v*/*v*) glycerol, 1 μM pepatatin, 2 μM leupeptin, 20 mM β-glycerophosphate, 20 mM NaF, 2 mM Na-ortovanodate, and 50 mM HEPES (pH 7.8)], kept on ice for 30 min, and centrifuged at 14,000× *g* for 10 min. The supernatant containing nuclear proteins was collected and stored at −80 °C in aliquots until use. The protein concentration was measured by the bicinchonic acid (BCA) assay method using bovine serum albumin (BSA) as a standard.

### 3.4. Western Blot

Western blotting was carried out as described previously and lysed samples were boiled for 5 min with gel-loading buffer [0.125 M Tris-HCl, pH 6.8, 4% SDS, 10% 2-mercaptoethanol, and 0.2% bromophenol blue] at a ratio of 1:1. Equal amounts of protein were separated by sodium dodecyl sulfate-polyacrylamide gel electrophoresis (SDS-PAGE) using 6–17% acrylamide gels. The gels were subsequently transferred onto Immobilon-P transfer membrane (Millipore Corp, Bedford, MA, USA). The membranes were immediately placed in blocking buffer [5% non-fat dry milk in TBS-Tween (TBS-T) buffer containing 10 mM Tris, 100 mM NaCl, and 0.1% Tween 20, pH 7.5] at room temperature for 1 h. The membrane was washed in TBS-T buffer for 30 min and incubated with the appropriate specific primary antibodies (1:1000 dilution) at 4 °C overnight. After 30 min washing in TBS-T buffer, the membrane was incubated with a secondary antibody (1:10,000 dilution) at room temperature for 1 h. Then, after washing in TBS-T buffer for 40 min, antibody labeling was detected using ECL per the manufacturer’s instruction and exposed to radiographic film.

### 3.5. Biochemical Analysis

Blood samples were collected from each group of rat and mice. Different kits were used, following manufacturer instructions, to determine the concentrations of various metabolites in serum: insulin (Shibayagi, Shibukawa, Japan) and glucose (Shinyang, Seoul, South Korea). Serum IL-1β was measured using the Luminex multiplex analysis system (Millipore, Billerica, MA, USA).

### 3.6. Glucose Tolerance Test

Mice were fasted for 18 h, followed by intraperitoneal injection of glucose (2 g/kg). Blood glucose levels were measured before and after the glucose injection by using a Glucometer Elite meter (Bayer, Whippany, NJ, USA).

### 3.7. In Silico Protein-Ligand Docking Simulation

Docking simulation is a computer simulation technique that is used to model the interaction between nuclear hormone receptors and their ligands. Among the many tools available for in silico protein-ligand docking, AutoDock4.2 is the most commonly used because of its automated docking capabilities. Ligand docking was performed using a set of predefined three-dimensional (3D) grids of the target protein and a systemic search technique. To prepare for the docking procedure, the following procedures were performed: (1) two-dimensional (2D) structures were converted into 3D structures; (2) charges were calculated; and (3) hydrogen atoms were added using the ChemOffice program (http://www.cambridgesoft.com).

### 3.8. Statistical Analysis

To analyze differences among three or more groups a one-way analysis of variance (ANOVA) was used. Differences in the means of individual groups were assessed by Bonferroni’s *post hoc* test. Student’s *t*-test was used to analyze differences between the two groups. Results are expressed as means ± S.E.M. *p* values of < 0.05 were considered statistically significant. Analyses were performed using GraphPad Prism 5 (GraphPad Software, La Jolla, CA, USA).

## 4. Conclusions

Our data showed that MLB potentially activated PPARβ/δ, which has been known to suppress ER stress and improve insulin sensitivity. However, it was not directly examined in our study whether or not the MLB-mediated amelioration of aging or obesity-induced ER stress, inflammasome formation, and disrupted insulin signaling are mediated through activating PPARβ/δ or through different mechanisms independent of PPARβ/δ. Studies have shown that MLB has free-radical scavenging activities [37]. A decrease in oxidative stress is closely related to ameliorating insulin signaling and reduced ER stress independent of PPARβ/δ [38]. On the other hand, other studies showed that PPARβ/δ agonist notably decreased insulin resistance, ER stress, and inflammasome [31,32,33,34]. Furthermore, a study showed that PPARβ/δ agonist treatment elevated insulin signaling and decreased inflammation in hepatocytes [38], indicating that the activation of PPARβ/δ contributes to the MLB-mediated beneficial effects on the liver. Therefore, it is assumed that both PPARβ/δ-dependent and -independent mechanisms likely contribute to the increased insulin signaling as well as the decrease in ER stress and inflammasome formation by MLB. Thus, MLB may be used as a pharmaceutical agent that ameliorates hepatic insulin sensitivity and metabolic syndrome derived from aging and obesity.

## Figures and Tables

**Table 1:** Summary of aging, obesity, and MLB actions in the liver.

Parameters	Signaling Pathway	Young & Chow	Old & HFD	MLB
ATF6α	ER stress	–	↑	↓
Caspase-1	Inflammasome	–	↑	↓
FoxO1	Insulin signaling & Gluconeogenesis	–	↑	↓
IL-1β	Inflammasome	–	↑	↓
NLRP3	Inflammasome	–	↑	↓
p-Akt (Ser473)	Insulin signaling	–	↓	↑
PEPCK	Gluconeogenesis	–	↑	↓
p-FoxO1 (Ser256)	Insulin signaling & Gluconeogenesis	–	↓	↑
p-FoxO1 (Thr24)	Insulin signaling & Gluconeogenesis	–	↓	↑
p-IRE	ER stress	–	↑	↓
p-IRS-1 (Ser307)	Insulin signaling	–	↑	↓
p-IRS-1 (Tyr632)	Insulin signaling	–	↓	↑
p-JNK	ER stress & Insulin resistance	–	↑	↓
PPARβ/δ	Multi-functional transcription factor	–	↓	↑
p-PERK	ER stress	–	↑	↓

–, the control; ↑, increased compared with the control; ↓ decreased compared with the control.

## Supplementary Materials

The following is available online, Figure S1: The body weight was measured at the end of the experiments to check whether HFD feeding or biological aging increase body weight as expected.
