# Supplementary material for: Mechanism of Action of Magnesium Lithospermate B against Aging and Obesity-Induced ER Stress, Insulin Resistance, and Inflammsome Formation in the Liver
Source: Molecules. 2018 Aug 21;23(9):2098. doi: 10.3390/molecules23092098 (PMC6225288; doi:10.3390/molecules23092098)
Supplement: Supplementary file 1 [file molecules-23-02098-s001.pdf]

Supplmentary Figure S1.

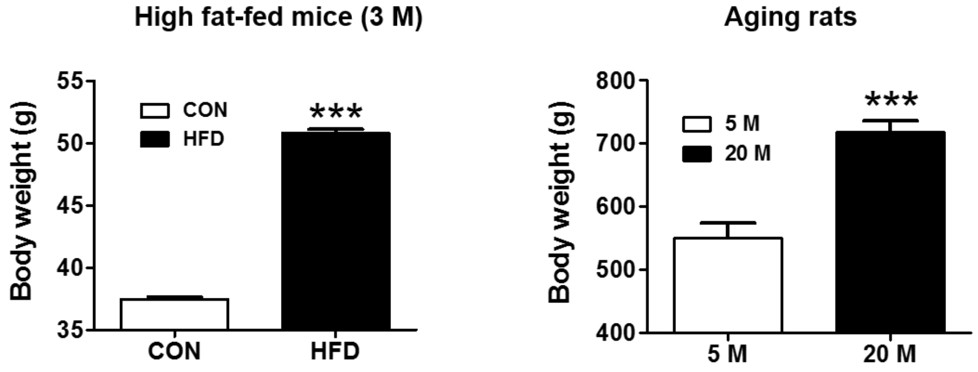

Supplmentary Figure S1. The body weight was measured at the end of the experiments to check whether HFD feeding or biological aging increase body weight as expected.
